# Reduced Expression of CLEC4G in Neurons Is Associated with Alzheimer’s Disease

**DOI:** 10.3390/ijms25094621

**Published:** 2024-04-24

**Authors:** Xinwei Feng, Fangfang Qi, Yuying Huang, Ge Zhang, Wenbin Deng

**Affiliations:** 1 School of Pharmaceutical Sciences (Shenzhen), Shenzhen Campus of Sun Yat-sen University, Shenzhen 510631, China; 2 Department of Neurology, Mayo Clinic, Rochester, MN 55901, USA; 3 Department of Anatomy and Physiology, Zhongshan School of Medicine, Sun Yat-sen University, Guangzhou 510080, China; 4 Department of Microbial and Biochemical Pharmacy, School of Pharmaceutical Sciences, Sun Yat-sen University, Guangzhou 510006, China

**Keywords:** CLEC4G, Alzheimer’s disease, neuronal cell, BACE1, APP/PS1 transgenic mice

## Abstract

CLEC4G, a glycan-binding receptor, has previously been demonstrated to inhibit Aβ generation, yet its brain localization and functions in Alzheimer’s disease (AD) are not clear. We explored the localization, function, and regulatory network of CLEC4G via experiments and analysis of RNA-seq databases. CLEC4G transcripts and proteins were identified in brain tissues, with the highest expression observed in neurons. Notably, AD was associated with reduced levels of CLEC4G transcripts. Bioinformatic analyses revealed interactions between CLEC4G and relevant genes such as BACE1, NPC1, PILRA, TYROBP, MGAT1, and MGAT3, all displaying a negative correlation trend. We further identified the upstream transcriptional regulators NR2F6 and XRCC4 for CLEC4G and confirmed a decrease in CLEC4G expression in APP/PS1 transgenic mice. This study highlights the role of CLEC4G in protecting against AD progression and the significance of CLEC4G for AD research and management.

## 1. Introduction

Alzheimer’s disease (AD) is a neurodegenerative disorder severely affecting cognitive functions, leading to reduced memory, diminished thinking capability, and impaired daily functioning. As one of the deadliest and most burdensome diseases globally, the pathogenesis of AD has attracted widespread attention [1]. β-site amyloid precursor protein-cleaving enzyme 1 (BACE1) serves as a central molecule, cleaving amyloid precursor protein (APP) to generate toxic amyloid-β (Aβ) peptides, leading to Aβ peptide aggregation and plaque formation in the brain, thereby initiating the progression of AD [2,3].

CLEC4G (C-type lectin domain family 4 member G, also known as LSECtin), as a C-type lectin, was initially identified in sinusoidal endothelial cells in the human liver and lymph nodes [4,5]. While CLEC4G plays a crucial role in cell adhesion and pathogen recognition [6,7,8], its role in the nervous system has remained insufficiently elucidated. Recent studies have discovered an interaction between CLEC4G and BACE1 in neuronal cell lines, suggesting a potential role of CLEC4G in inhibiting BACE1-mediated Aβ generation [9]. However, despite previous studies highlighting the potential significance of CLEC4G, its expression levels in AD brain tissue and its potential association with AD remain unclear.

Therefore, this study aims to explore the correlation between CLEC4G and AD by analyzing the expression levels of CLEC4G between AD patients and non-demented individuals, combining single-cell RNA sequencing data and large-scale RNA sequencing datasets. We will also examine the cellular sources and expression levels of CLEC4G in human and APP/PS1 model mouse brain tissues to fill the knowledge gap regarding the role of CLEC4G in the pathogenesis of AD. Our study will contribute to a deeper understanding of the role of CLEC4G in the pathogenesis of AD, providing a theoretical basis and important reference for the future development of novel therapeutic strategies.

## 2. Results

### 2.1. High Levels of CLEC4G Are Expressed in Both Mouse and Human Brain Tissues

**Figure 1:**
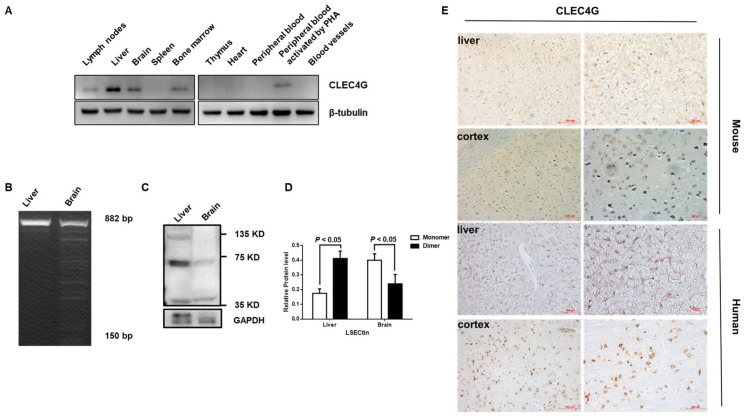
CLEC4G expression in mouse and human brain tissues. (**A**) CLEC4G protein was detected using Western blots in different mouse tissues. (**B**) The complete coding sequence (882 bp) of CLEC4G was detected via RT–PCR in mouse liver and brain tissues and then analyzed using electrophoresis. (**C**) Western blotting analysis and grayscale analysis of mouse brain and liver tissue homogenates with LSECtin antibody. (**D**) Statistical analysis was conducted on staining intensities, with each experiment performed in triplicate. The intensities of the bands were assessed using Image J software 1.54 and GraphPad Prism 9.4.0. All values are means ± S.D. (error bars). (**E**) Expression of CLEC4G protein in paraffin sections of human and mouse brain tissues.

We analyzed the expression levels of CLEC4G in mouse tissues using Western blotting. As shown in Figure 1A, CLEC4G was detected in the lymph nodes, liver, brain, and bone marrow but not in the spleen, thymus, blood vessels, and heart of wild-type mice. CLEC4G mRNA was also detected in the PHA-activated peripheral blood (PHA, Phytohemagglutinin) but not in the non-stimulated peripheral blood of mice.

Interestingly, in addition to the full coding region of CLEC4G (882 bp), we also observed a series of shorter amplicon bands in brain tissues. These fragments were identified as different splice variants of CLEC4G via DNA sequencing (Figure 1B). Moreover, a higher level of monomer than dimer forms of CLEC4G was detected in the brain, whereas higher dimer form levels were detected in the liver using Western blotting analysis (Figure 1C,D). Immunohistochemical analysis showed that strongly positive staining of CLEC4G was concentrated in the cortex and hippocampus in the mouse brain tissues. Notably, CLEC4G-positive cells were mainly located in the cytoplasm in both human and mouse brain tissues. However, CLEC4G was detected mainly on the membranes of positively stained liver cells (Figure 1E).

Our results demonstrated similar expression levels of CLEC4G in brain and liver tissues. However, CLEC4G in the brain has different splice variant RNAs and major monomer forms and is found in the cytoplasm.

### 2.2. CLEC4G Is Mainly Expressed in Neurons

**Figure 2:**
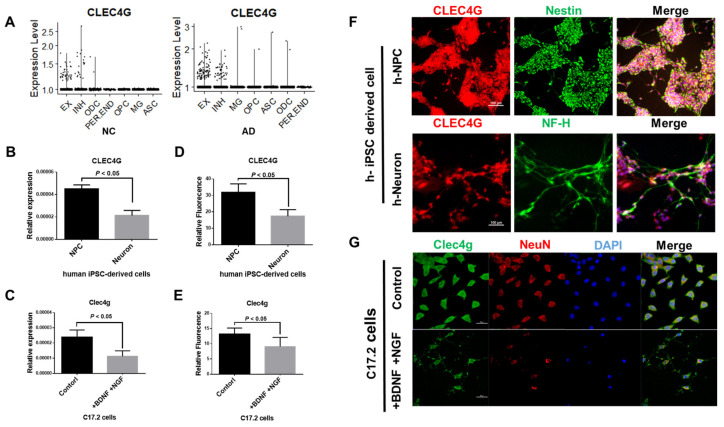
LSECtin protein expression in iPSC-derived neuronal cells and C17.2 cells. (**A**) Single-cell RNA-seq data revealing the expression of CLEC4G in various human brain cells (ASC: astrocyte; EX: excitatory neuron; INH: inhibitory neuron; MG: microglia; ODC: oligodendrocyte; OPC: oligodendrocyte progenitor cell; PER. END: pericyte/endothelial) (data source: A, GSE174367). (**B**,**C**) RNA expression levels of CLEC4G in (**B**) hiPSC-derived NPCs and neurons or (**C**) undifferentiated and differentiated C17.2 cell (n = 3). (**F**,**G**) Immunofluorescence of CLEC4G in (**F**) hiPSC-derived NPCs and neurons or (**G**) undifferentiated and differentiated C17.2 cells. The relative fluorescence intensity of these cells was quantified as (**D**,**E**) (n = 3). Scale bars: 100 μm (**top right**) and 50 μm (**bottom right**). Student’s *t*-test was used to determine differences between two groups.

Subsequently, we analyzed the expression of CLEC4G in the brains of AD patients based on single-cell RNA-seq (scRNA-seq) data. Figure 2A shows that CLEC4G is predominantly expressed in neurons, including excitatory and inhibitory neurons, in the brain cells of healthy individuals and AD patients. Then, we detected the expression of CLEC4G in the mouse neural progenitor cell line C17.2 as well as in human iPSC-derived neuronal cells. The human iPSC-derived neural progenitor cells (NPCs) showed higher levels of CLEC4G than neurons in both RT–PCR and immunofluorescence analysis (Figure 2B–G). Similarly, undifferentiated C17.2 cells had higher expression of CLEC4G than BDNF- and NGF-induced differentiated C17.2 cells (Figure 2B–G). Notably, fluorescence staining showed that CLEC4G was located in both the cytoplasm and membranes of these cells.

In summary, our results demonstrated that CLEC4G was strongly expressed in the cytoplasm and membranes of neurons, and higher expression of CLEC4G was observed in the undifferentiated neurons or NPCs.

### 2.3. Reduced Expression of CLEC4G in Patients with AD and APP/PS1 Mice with Increasing Age

**Figure 3:**
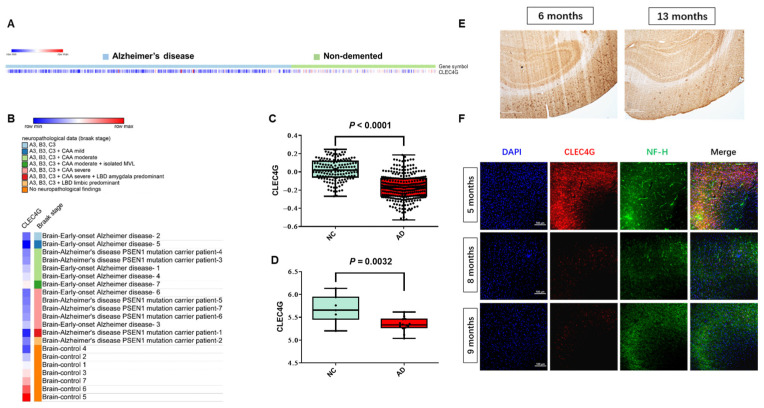
Reduced expression levels of LSECtin protein in patients with Alzheimer’s disease. (**A**,**B**) CLEC4G Bulk RNA-seq data reveal the expression of CLEC4G in non-demented people and Alzheimer’s disease patients. (Data source: (**A**), GSE33000; (**B**), GSE39420.) (**C**) CLEC4G expression levels were significantly decreased in AD patients based on bulk RNA-seq data (NC, normal control, n = 157; AD, Alzheimer’s disease, n = 310; data source: GSE33000). (**D**) CLEC4G expression levels significantly decreased in AD patients based on bulk RNA-seq data (NC, normal control, n = 7; AD, Alzheimer’s disease, n = 14; data source: GSE39420). (**E**) Immunohistochemistry analysis of CLEC4G in the APP/PS1 mouse model. Scale bar: 500 μm. (**F**) Immunofluorescence analysis of CLEC4G in the APP/PS1 mouse model (red: CLEC4G; green: neurofilament H). Scale bar: 100 μm.

To investigate the possible roles of CLEC4G in AD, we first analyzed the expression levels of CLEC4G in brain tissue from AD patients and non-demented individuals (NC) using bulk RNA-seq data from the GEO database. The samples of two datasets, GSE33000 and GSE39420, were both from fresh-frozen postmortem brain tissues, and the mRNA levels of CLEC4G were high in the NC/Non-demented group (Figure 3A,B). Compared to the NC group (n = 157; n = 7, respectively), the level of CLEC4G mRNA was significantly lower in the AD group (n = 310; n = 14, respectively) (*p* < 0.01; *p* = 0.0032) (Figure 3C,D).

Next, we validated the protein expression of CLEC4G in APP/PS1 model mice. The expression of CLEC4G was significantly lower in the cortical region of 13-month-old mice than in that of 6-month-old mice according to immunohistochemical analysis (Figure 3E). Similarly, the expression of CLEC4G in the cortical area was decreased with increasing age in APP/PS1 mice, as shown via immunofluorescence analysis (Figure 3F).

It is well known that as AD progresses, there is typically a decrease in the number of neurons in the brain [10,11]. The observed decrease in CLEC4G expression in AD could be due to the reduction in neuron numbers. To address this question, we utilized single-cell data from GSE174367, which contains 38,676 nuclei in late-stage AD and 22,796 nuclei in non-demented controls. A UMAP visualization of excitatory and inhibitory neurons in the AD and control groups is shown in Figure S1A. We analyzed the proportions of CLEC4G-positive cells in the AD and control groups. Specifically, the percentages of CLEC4G-positive cells were found to be 1.44% in the AD group and 1.80% in the control group (Figure S1B). We then analyzed CLEC4G expression levels in these positive cells and found that the control group exhibited significantly higher expression levels compared to the AD group (*p* = 0.0186, Figure S1C), a finding consistent with the results obtained from bulk RNA-seq analysis. This indicates that the decreased expression of CLEC4G in AD is not due to a reduction in neuron numbers.

These data suggest that AD patients have a lower level of CLEC4G than non-demented individuals, and these reduced CLEC4G levels may be closely related to AD progression.

### 2.4. Functional Analysis of CLEC4G Protein in Neurons

**Figure 4:**
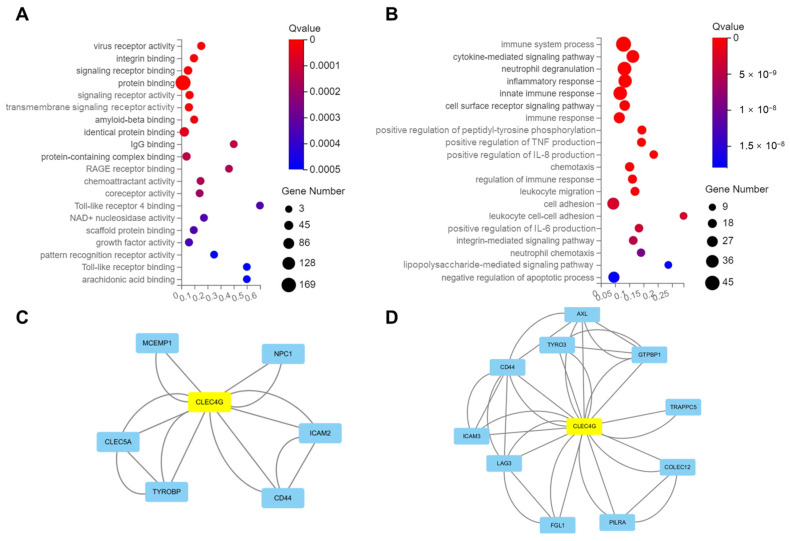
Functional analysis of CLEC4G protein. (**A**) GO molecular function enrichment analysis of CLEC4G-related genes. (**B**) GO biological process enrichment analysis of CLEC4G-related genes. (**C**,**D**) Protein–protein interaction analysis of CLEC4G in (**C**) CLEC4G-related genes and (**D**) a genome-wide scale.

To further analyze the function of CLEC4G in the brain, we performed GO-molecular function and GO-biological process enrichment analyses of CLEC4G-related genes. As shown in Figure 4A,B, CLEC4G was found to be closely related to cell adhesion and inflammation-related pathways. Additional analysis indicated that CLEC4G was involved in amyloid-beta binding.

Then, we analyzed the regulatory relationships of CLEC4G-related genes via protein–protein interaction (PPI) analysis and identified six neighboring genes of CLEC4G, including NPC1 (intracellular cholesterol transporter 1), TYROBP (transmembrane immune adaptor), CD44, CLEC5A, ICAM2 (intercellular adhesion molecule 2), and MCEMP1 (mast cell expressed membrane protein 1) (Figure 4C). Most of these genes play a role in cell adhesion, the regulation of inflammation, and immune response, except NPC1, which was reported to be linked to perturbed cholesterol homeostasis in AD [12].

Next, we identified the genes with possible interactions with CLEC4G using the String database. According to the confidence score, the top 10 genes were CD44, AXL (AXL Receptor Tyrosine Kinase), ICAM3, TYRO3 (TYRO3 Protein Tyrosine Kinase), PILRA (Paired Immunoglobin-Like Type 2 Receptor Alpha), FGL1 (Fibrinogen-Like 1), LAG3 (Lymphocyte-Activating 3), TRAPPC5 (Trafficking Protein Particle Complex Subunit 5), COLEC12 (Collectin Subfamily Member 12), and GTPBP1 (GTP-Binding Protein 1) (Figure 4D). These genes are related to immune regulation, cell migration, and vesicle transport.

These bioinformatic analyses demonstrate that CLEC4G is closely related to cell adhesion, extracellular signaling, and inflammatory and immune-related pathways. CLEC4G may be involved in the process of AD through its interaction with NPC1, PILRA, and TYROBP.

### 2.5. CLEC4G Protein Plays a Positive Role in AD Progression

**Figure 5:**
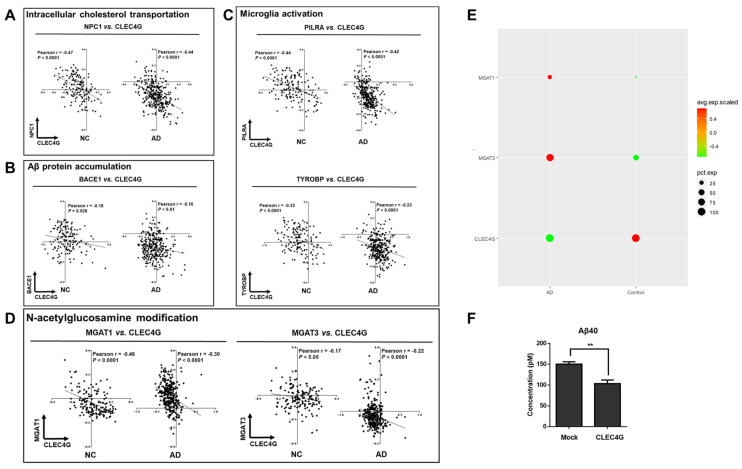
CLEC4G plays a positive role in AD pathogenesis. (**A**) RNA co-expression analysis between CLEC4G and NPC1. NPC1, NPC intracellular cholesterol transporter 1. (**B**) RNA co-expression analysis between CLEC4G and BACE1. BACE1, beta-secretase 1. (**C**) RNA co-expression analysis between CLEC4G and PILRA or TYROBP. The solid line indicates linear fit. Pearson’s correlation coefficient (r) and *p* values indicate the significance of correlation. (**D**) RNA co-expression analysis between CLEC4G and MGAT1 or MGAT3. The solid line indicates linear fit. Pearson’s correlation coefficient (r) and *p* values indicate the significance of correlation. (**E**) Single-cell data analysis revealing the expression levels of CLEC4G, MGAT1, and MGAT3 in CLEC4G-positive cells from AD and control groups. (**F**) hiPSC-derived neurons were subjected to transfection using a plasmid carrying the human CLEC4G gene or an empty plasmid (mock). Subsequently, the quantity of Aβ40 present in the culture medium was assessed (n = 3). The graph depicts the mean values along with the standard error of the means (S.E.M.s), analyzed using Student’s *t*-test. (NPC1, NPC Intracellular Cholesterol Transporter 1; PILRA, Paired Immunoglobin Like Type 2 Receptor Alpha; TYROBP, Transmembrane Immune Signaling Adaptor TYROBP; BACE1, Beta-Secretase 1). ** *p* < 0.01.

The correlation between CLEC4G and the three genes NPC1, PILRA, and TYROBP in both the NC and AD groups was examined via RNA co-expression analysis. CLEC4G exhibited a significant negative correlation with NPC1 (R = −0.47, *p* < 0.0001; R = −0.44, *p* < 0.0001), PILRA (R = −0.044, *p* < 0.0001; R = −0.42, *p* < 0.0001), and TYROBP (R = −0.32, *p* < 0.0001; R = −0.23, *p* < 0.0001) in both NC and AD (Figure 5A–C).

The NPC1 protein facilitates the transportation of cholesterol from lysosomes to other organelles [12,13]. Studies have indicated that there is elevated NPC1 expression in Alzheimer’s disease (AD), a phenomenon associated with disrupted cholesterol homeostasis in AD [14,15]. Thus, the negative correlation between CLEC4G and NPC1 expression suggests a positive role of CLEC4G in maintaining cholesterol homeostasis in AD. PILRA and TYROBP are highly expressed in AD and involved in the activation of microglial cells [16,17]. They are considered risk genes for AD [18]. The negative correlations between CLEC4G and PILRA expression, as well as between CLEC4G and TYROBP expression, suggest that CLEC4G may play a protective role in AD by influencing microglial activation.

We also analyzed the correlation between CLEC4G and BACE1 expression in both NC and AD patients. As shown in Figure 5B, CLEC4G exhibited a significantly negative correlation with BACE1 in both the AD group (r = −0.16, *p* < 0.01) and the NC group (r = −0.18, *p* = 0.028). Furthermore, we overexpressed CLEC4G in iPSC-derived cortical neurons. The amount of Aβ40 in the culture media was decreased in CLEC4G-overexpressing cells, as determined via ELISA analysis (*p* < 0.01) (Figure 5F).

It has been reported that abnormalities in glycosylation may be involved in the pathogenesis of AD [19,20]. Several studies have identified abnormalities of glycosylation in the brains of AD patients, including abnormalities of glycosylation related to MagT1 and Mgat3. Upregulation of MGAT1 is involved in complex N-linked glycan formation [21]. Loss of GnT-III (Mgat3) reduces BACE1-mediated Aβ generation, leading to the amelioration of AD pathology [9]. Our results suggest that there is a close relationship between CLEC4G and Mgat1- and Mgat3-mediated glycosylation modifications. As shown in Figure 5D, CLEC4G exhibited a significant negative correlation with MGAT1 in both the AD group (r = −0.46, *p* < 0.0001) and the NC group (r = −0.30, *p* < 0.0001). CLEC4G also exhibited a significant negative correlation with MGAT3 in both the AD group (r = −0.17, *p* < 0.05) and the NC group (r = −0.22, *p* < 0.0001). Single-cell data further demonstrate that in AD patients, MGAT1 and MGAT3 are highly expressed, while CLEC4G is expressed at a lower level. In contrast, MGAT1 and MGAT3 are expressed at lower levels, while CLEC4G was expressed at a higher level in the control group (Figure 5E).

These results suggest that the CLEC4G protein might protect individuals from AD progression by playing roles in intracellular cholesterol transport, microglia activation, and APP protein cleavage.

### 2.6. Regulatory Relationships of the CLEC4G Gene in Neurons

**Figure 6:**
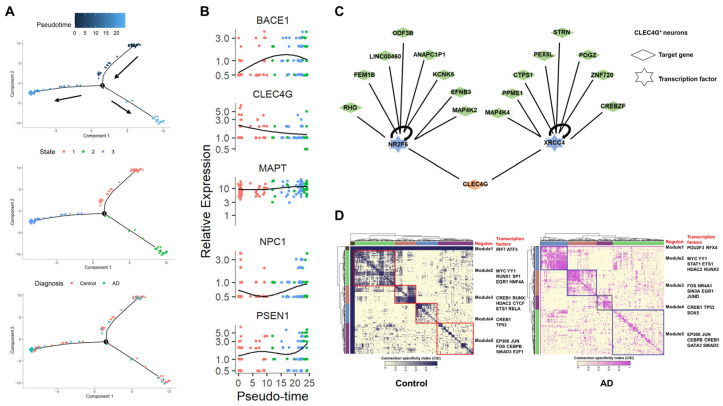
Single-cell data analysis reveals the regulatory relationships of the CLEC4G gene in neurons. (**A**) Monocle 2 trajectory plot showing developmental trajectories and state dynamics of CLEC4G^+^ neurons. The arrows indicate the direction of cell development in the pseudotime analysis. (**B**) Five representative genes exhibiting diverse expression patterns during the differentiation of CLEC4G^+^ neurons: CLEC4G, BACE1, MAPT, NPC1, and PSEN1. (**C**) SCENIC analysis reveals upstream transcription factors regulating CLEC4G in neurons. (**D**) Identification of transcription factor modules based on regulon activities in control and AD CLEC4G^+^ neurons. Square represents different transcription factor modules.

To delve deeper into the regulation network of CLEC4G, we conducted a comprehensive analysis of brain single-cell data, which included information on both healthy individuals and AD patients. Cell trajectory analysis simulated the developmental process of control neurons transitioning to AD neurons (Figure 6A). The results indicated a gradual decrease in the expression levels of the CLEC4G gene during this process, while genes such as BACE1, PSEN1, and NPC1 showed a pronounced upward trend. This outcome suggests that CLEC4G expression decreases as AD progresses, corroborating the results of the bulk RNA-seq analysis shown in Figure 5.

Subsequently, we employed Single-Cell Regulatory Network Inference and Clustering (SCENIC) [22] to investigate the regulatory relationships of the CLEC4G gene in neuronal cells (Figure 6C). The results revealed that CLEC4G is primarily regulated by the upstream transcription factors NR2F6 and XRCC4. We then examined whether the significant differences in CLEC4G expression between healthy individuals and AD patients would lead to changes in transcription factor modules within neuronal cells. Based on SCENIC data, we identified a gene-regulatory network that was grouped into five major modules across the control and AD neuronal cells. This analysis revealed the aggregation of transcription factors into different functional modules within the same cell population. It became evident that the transcription factor regulation modules of neuronal cells with high CLEC4G expression (control group) were noticeably distinct from those of neuronal cells with low CLEC4G expression (AD group), implying a significant difference in the states of these two cell types.

## 3. Discussion

In this study, we demonstrated that CLEC4G is strongly expressed in both the mouse and human brains and is mainly located on the cell membrane and in the cytoplasm of neurons. In contrast to CLEC4G in the liver—which is mainly located on the cell membrane, with dimer forms serving as receptors that play the role of pathogen recognition [6,23]—CLEC4G in the brain is located in the cytoplasm in monomer forms. These differences suggest that CLEC4G might play roles in nerve cells both intracellularly and extracellularly.

We found that CLEC4G expression was significantly higher in non-demented individuals than in AD patients. The scRNA-seq data also revealed a gradual decrease in CLEC4G levels during the transition from healthy neurons to AD neurons, a finding validated in the APP/PS1 transgenic mouse model (Figure 3E,F). The enrichment analysis of CLEC4G-related genes identified a close association with the binding of Aβ proteins. Previous studies have suggested that mouse CLEC4G functions as an inhibitor of BACE1 [9]. Our research supports this conclusion, as both bulk-RNA-seq and scRNA-seq analyses indicate an inverse correlation between CLEC4G expression and BACE1 in neurons (Figure 5 and Figure 6B). Subsequently, our experiments demonstrated that overexpression of CLEC4G significantly reduces the levels of Aβ40 in human neurons (Figure 5F). Our results also indicate that CLEC4G has a significant negative correlation with BACE1 instead of MAPT (encoding tau protein) (Figure 6B). This suggests that CLEC4G may primarily influence the Aβ protein rather than tau protein. These findings suggest that CLEC4G may play a role in protecting individuals from AD progression by inhibiting the generation of Aβ.

The CLEC4G gene encodes a sugar-binding receptor with high affinity for GlcNAc (N-acetylglucosamine) [24]. N-glycan structures play a crucial role in various physiological and pathological events, including cancer and AD progression [20,25]. Interestingly, N-acetylglucosamine is a unique sugar modification most highly expressed in the nervous system [26]. It has been reported that the levels of N-acetylglucosamine on BACE1 are elevated in AD patients, and removal of N-acetylglucosamine has been shown to ameliorate AD pathology [27]. Our results reveal a significant negative correlation between CLEC4G and MGAT3 (N-acetylglucosamine synthase) in both the AD group (r = −0.17, *p* < 0.05) and the NC group (r = −0.22, *p* < 0.0001). Additionally, immunohistochemical results demonstrate that CLEC4G expression decreases with the progression of AD (Figure 3E). These findings suggest that a reduction in CLEC4G may impair neuronal function in AD by limiting the clearance of N-acetylglucosamine.

Our data also indicate that CLEC4G interacts with PILRA and TYROBP proteins. The expression levels of CLEC4G showed a significant negative correlation with PILRA and TYROBP. PILRA has been reported to be one of the receptors for CLEC4G, and it is also one of the risk genes for AD [28,29], while TYROBP is a common adapter for many immune cell receptors. In the brain, PILRA and TYROBP are primarily expressed in microglial cells and associated with microglial cell inflammation [18,30]. This suggests that CLEC4G may influence AD progression by modulating microglial cell activation. Recent studies indicate that the activation of the TREM-SYK (TREM, myeloid cell-expressed activating receptor; SYK, spleen-associated tyrosine kinase) pathway is crucial for inducing the activity of functional microglial cells. Treatment of TREM2R47H mutant mice with anti-CLEC7A antibodies systemically rescued microglial activation and enhanced Aβ phagocytosis [31]. Another study suggested that LSECtin induces SYK activation by binding to DAP12 (DNAX-activating protein 12, encoded by the TYROBP gene) [32] (Zhao et al., 2016). It can be speculated that high expression of CLEC4G may contribute to SYK activation, thereby promoting microglial cell activation.

The immune system plays a profound role in the progression of Alzheimer’s disease (AD) [33]. CLEC4G is a C-type lectin of the immunoglobulin superfamily closely associated with the immune response of neurons. CLEC4G on neuronal cells may serve as a messenger communicating with microglial cells, thereby modulating the activation of immune cells. In AD, the reduced expression of CLEC4G may lead to a disruption in communication between neurons and microglial cells, further resulting in abnormal immune microenvironments or immune response imbalance. This hypothesis requires further validation.

Based on detailed scRNA-seq analyses of the regulatory network of CLEC4G in neuronal cells, we identified two upstream transcriptional regulators of CLEC4G through SCENIC analysis, namely, NR2F6 and XRCC4 (Figure 6C, Figure S3). It has been reported that the deletion of Nr2f6 (Ear2) leads to defects in early memory and learning in the APP/PS1 mouse AD model [34]. NR2F6 levels are decreased in AD (Figure S2), which may contribute to AD progression and could be one of the reasons for the decrease in CLEC4G expression. In summary, our results suggest a beneficial role of CLEC4G in protecting individuals from AD progression.

Our study highlights the expression of CLEC4G in the brain, predominantly on neurons, and its potential role in Alzheimer’s disease (AD). Furthermore, we observed significant differences in CLEC4G expression levels between AD patients and healthy individuals, with markedly reduced expression in AD patients. This conclusion was supported by bulk RNA-seq, single-cell RNA-seq, and animal slice data analyses. Through pseudo-time analysis, we simulated the transition from healthy neurons to AD neurons and found a gradual decrease in CLEC4G levels, providing evidence from a developmental biology perspective. Some conclusions derived from bioinformatics analyses in this study need further experimental validation, which is a limitation of our research. For example, it is unclear whether CLEC4G binds to BACE1 through N-acetylglucosamine and whether NR2F6 is an upstream transcription factor of CLEC4G, among others.

In conclusion, CLEC4G may represent a potential novel target in AD research and management. Further studies are needed to confirm and elucidate the exact mechanisms of CLEC4G in the progression of AD. Future research directions may include investigating whether increasing CLEC4G expression can reverse the AD phenotype in cellular or animal models and exploring how neurons regulate microglial cells through CLEC4G.

## 4. Materials and Methods

### 4.1. Cell Lines

Human iPSCs were maintained on Matrigel-coated plates in mTeSR1 medium (STEMCELL Technologies, Vancouver, BC, Canada, 85850), following a previously established protocol [35]. When the cell confluence reached approximately 20%, the medium was replaced with NIM1, and cells were cultured for 3 days. Subsequently, the medium was changed to NIM2 for an additional 5 days, conducting medium renewal every two days. On day 8, cells were dissociated and passaged using ACCUTASE (Sigma, St. Louis, MO, USA, A6964); then, they transferred to NSMM medium for continued culture. For neuronal differentiation, neural progenitor cells (NPCs) were cultured in DM medium for 14 days. The specific formulations of NIM1, NIM2, NSMM, and DM can be found in Supplementary Table S2.

The C17.2 cell line (mouse multipotent neural progenitor cells) was cultured in DMEM (Invitrogen, Carlsbad, CA, USA) supplemented with 10% fetal bovine serum (FBS). To induce differentiation, the cells were treated with 50 ng/mL of BDNF (Peprotech, Berkeley, NJ, USA, AF-450-02) and 50 ng/mL of NGF (Peprotech, Berkeley, NJ, USA, 450-01). Culturing of all cell lines was conducted in standard humidified incubators at 37 °C and with 5% CO_2_.

### 4.2. RT-PCR

The extraction of total RNA from cell lines and mouse tissues was carried out using the Trizol reagent (Invitrogen, Carlsbad, CA, USA) in accordance with the manufacturer’s instructions. Reverse transcription of 2 μg of total RNA was conducted using SuperScript II reverse transcriptase. The following primers were used in the PCR reaction. CLEC4G coding sequence: forward 5′-AGTCCTTTGGGCTGTGATTCT-3′ and reverse 5′-AGGCGTTTGTCCTCAGCAG-3′; reference gene GAPDH: forward 5′-GCACCGTCAAGGCTGAGAAC-3′ and reverse 5′-TGGTGAAGACGCCAGTGGA-3′. Clec4g coding sequence: forward 5′-ACTGGTGAATACAACAAGCTGG-3′ and reverse 5′-ACTGGACAGTAGGGTGCTCAG-3′; and reference gene Actb (β-actin): forward 5′-GTGACGTTGACATCCGTAAAGA-3′ and reverse 5′-GCCGGACTCATCGTACTCC-3′.

### 4.3. Western Blot

Protein extraction was performed using a lysis buffer supplemented with protease inhibitor (Beyotime, Wuhan, China) for 20 min. After centrifugation at 4 °C for 10 min at 12,000× *g*, the supernatant was collected. The protein concentration was quantified using the BCA Kit (Thermo Scientific, Wilmington, MA, USA, 23227). Afterward, the protein was mixed with protein loading buffer (Beyotime, China) and heated at 100 °C for 15 min. Equivalent amounts of protein samples were then prepared in SDS sample buffer, followed by separation through 10% SDS-PAGE and a subsequent transfer onto polyvinylidene difluoride membranes. Following blocking with blocking buffer (Beyotime, Wuhan, China), primary antibodies targeting CLEC4G (1:1000, Thermo Scientific, Wilmington, MA, USA, PA5-53116) were used to probe the membranes, followed by incubation with secondary antibody (Bioworld, Beijing, China) at a dilution ratio of 1:5000. Mouse β-tubulin levels served as a loading control, using a specific antibody (BS1842, Bioworld, Beijing, China) at a dilution ratio of 1:1000. ImageJ software was used to measure the intensity of the bands.

### 4.4. Immunohistochemistry

The brain paraffin sections of male APP/PS1 transgenic mice, aged 6 and 13 months, were provided by Prof. Rongbiao Pi. Seven sections were obtained from each age group. The paraffin-embedded tissue sections underwent dewaxing, rehydration, and rinsing. The tissue sections underwent antigen retrieval through heating at 100 °C for 20 min in a citrate solution (10 mmol/L, pH 6.0). To block endogenous peroxidase activity, the sections were immersed in a 3% H_2_O_2_ solution for 10 min. Subsequently, the sections were left to incubate overnight at 4 °C with the primary antibody CLEC4G Antibody (diluted 1:500, Thermo Scientific, Wilmington, MA, USA, PA5-53116). For negative control, PBS was used instead of the primary antibody. Following this, the sections were treated with an HRP-labeled secondary antibody (diluted 1:200, Bioworld, Beijing, China, BS13278) at room temperature for 120 min. Finally, the signal was visualized using DAB (3,3′-diaminobenzidine tetrahydrochloride), and all slides underwent counterstaining with hematoxylin.

### 4.5. Immunofluorescence

Frozen brain sections from 5-, 8-, and 9-month-old APP/PS1 transgenic mice were generously provided by Dr. Fangfang Qi. Six sections were obtained from each age group. Mouse brain tissues were embedded in OCT embedding medium, frozen, and then sectioned into 4 μm slices. For frozen sections, prior to staining, the sections were fixed in cold acetone at 4 °C for 10 min, followed by two washes with PBS for 5 min each. The endogenous peroxidase activity was inhibited via treatment with 3% H_2_O_2_ for 10 min in the dark, followed by two additional washes with PBS for 5 min each. Blocking solution was then applied and incubated at room temperature for 1 h. For cell fixation, a solution containing 4% paraformaldehyde (PFA) was applied, followed by permeabilization using 0.2% Triton X-100 for 15 min at room temperature. Subsequently, blocking was performed with 10% BSA for 30 min. The sections or cells were then incubated overnight at 4 °C with primary antibodies against CLEC4G (1:500, Thermo Scientific, Wilmington, MA, USA, PA5-53116), NeuN (1:250, Abcam, Boston, MA, USA, ab177487), Nestin (1:250, Abcam, Boston, MA, USA, ab18102), or Neurofilament-H (1:250, CST, Danvers, MA, USA, 55453). Following that, the cells or sections were incubated with the corresponding fluorescent secondary antibodies (diluted 1:1000, Boster, Wuhan, China) for 1 h at room temperature. Subsequently, the cell nuclei were visualized through DAPI staining. Imaging of the cells was performed at 40× magnification using the Zeiss LSM710 confocal microscope (Zeiss, Jena, Germany).

### 4.6. Animal Experiment

The animal experiments were conducted in accordance with ethical guidelines and approved protocols. Ethical clearance for the animal experiments was obtained from the Ethics Committee of Sun Yat-sen Memorial Hospital, Sun Yat-sen University. The animal use protocol was reviewed and approved by the Animal Ethical and Welfare Committee of Sun Yat-Sen Memorial Hospital, Sun Yat-sen University (Approval number: AP20220138). C57BL/6 mice were acquired from the Animal Experiment Center at Sun Yat-sen University. Various organ samples used in the experiments were sourced from C57BL/6 mice (3-month-old male mice, 6 in all). Phytohemagglutinin (PHA) was administered via tail vein injection at a dose of 2 mg/kg to activate peripheral blood, and samples were collected 24 h after administration for subsequent PCR and Western blot assays (3-month-old male mice, 6 in all). Human brain paraffin sections were provided by the Forensic Identification Center at Sun Yat-sen University, with informed consent obtained from the relatives of the human subjects.

### 4.7. Transfection

The human CLEC4G plasmid pcDNA3.1-mLestin was generously provided by Dr. Tang Li. Human iPSC-derived neuron cells were cultured in DMEM/F12 medium supplemented with 1x B27 and N2, 10 ng/mL of BDNF, and 10 ng/mL of NGF. Plasmid transfection was carried out using Lipofectamine 2000 reagent (Invitrogen, Carlsbad, CA, USA, 11668019), following the manufacturer’s instructions. Briefly, cells at approximately 50% confluence were transfected with 0.2 μg/cm^2^ of CLEC4G plasmid. The medium was changed six hours after transfection.

### 4.8. Aβ40 ELISA

Samples were obtained from the culture supernatant of iPSC-derived neuron cells. ELISA for Aβ40 was conducted utilizing the β-amyloid (40) ELISA kit (Wako, 294-62501, Tokyo, Japan), following the manufacturer’s protocol.

### 4.9. Data Resource and Description

The sequence data from both AD patients and non-demented individuals were acquired from the NCBI Gene Expression Omnibus (GEO) database. This included GEO series GSE33000 and GSE39420 for bulk RNA-seq data, as well as GSE174367 for scRNA-seq data. The platforms and samples associated with these GEO series are outlined in Table S1. Subsequently, the bulk RNA-seq and scRNA-seq data from all control subjects and AD patients were analyzed and compared.

### 4.10. Identification of CLEC4G-Related Genes

RNA-Seq data (GSE33000) of 310 AD and 157 non-demented samples were obtained from the official GEO website, and the edgeR package [36,37] was used to compare mRNA expression between AD and non-demented samples to identify differentially expressed genes (DEGs). A cut-off criterion of *p* < 0.05 and |logFC| > 1 was applied. The Spearman coefficients of DEGs and CLEC4G were calculated, and DEGs with a *p* < 0.05 were defined as CLEC4G-related genes.

### 4.11. GO Biological Process Enrichment

We utilized the Dr. TOM system provided by BGI Genomics Co., Ltd., Shenzhen, China (http://biosys.bgi.com, accessed on 1 June 2023), an online RNA data analysis tool, for conducting Gene Ontology (GO) enrichment analysis, covering biological process (BP) and molecular function (MF). A significance threshold of *p* < 0.05 was applied.

### 4.12. Protein–Protein Interaction (PPI) Network Construction

To predict CLEC4G interaction relationships, we used STRING (http://string-db.org, accessed on 1 April 2023) (version 11.5) [38] and conducted the analysis in two dimensions. First, for the differentially expressed genes (DEGs) between AD and non-demented samples, we uploaded CLEC4G-related genes to the STRING website and selected interactors that directly connect with CLEC4G based on a minimum required interaction score of 0.400 (medium confidence). Second, on a genome-wide scale, we uploaded only CLEC4G to the STRING website and selected the top 10 interactors based on a minimum required interaction score of 0.400. We inputted the resulting protein–protein interaction (PPI) pairs into Cytoscape software (version 3.7.1) [39] to construct a PPI network.

### 4.13. Gene Regulatory Network Analysis of CLEC4G Gene

#### 4.13.1. Trajectory Analysis

We employed the Monocle 2 package to establish developmental pseudo-time. The initial step involved converting raw counts from the Seurat object to the CellDataSet object using the importCDS function in Monocle. Subsequently, the differential GeneTest function within the Monocle 2 package was applied to identify ordering genes (with a q-value < 0.01), which were expected to provide valuable insights into cell ordering along the pseudo-time trajectory. Dimensional reduction clustering analysis was carried out utilizing the reduceDimension function, followed by trajectory inferencing performed using the orderCells function with default parameters. Subsequently, gene expression patterns were visualized using the plot_genes_in_pseudotime function to track changes across pseudo-time.

#### 4.13.2. SCENIC Analysis

A SCENIC analysis was performed, employing the motif database for GRNboost and RcisTarget, with default parameters. In summary, we employed the RcisTarget package to identify the enrichment of transcription factor (TF)-binding motifs within a designated gene list. Following this, we utilized the AUCell package to evaluate the activity of each regulon group in every individual cell. In order to evaluate the cell type specificity of each predicted regulon, a regulon specificity score was computed using the Jensen–Shannon divergence to measure the similarity between two sets of probability distributions.

### 4.14. Pearson’s Correlation Analysis

Correlations between NPC1 and CLEC4G, PILRA and CLEC4G, TYROBP and CLEC4G, and BACE1 and CLEC4G were calculated using Pearson’s correlation via IBM SPSS software 20.0 (SPSS, Inc., Chicago, IL, USA).

### 4.15. Statistical Analysis

All data were presented as the mean ± standard deviation (SD) from three independent experiments. Student’s *t*-test was employed to compare differences between two groups, whereas one-way ANOVA was utilized for comparisons involving three or more groups. Statistical analyses were conducted using SPSS 20.0 (SPSS, Inc., Chicago, IL, USA) on a Windows platform. A *p*-value below 0.05 was considered statistically significant.

## Data Availability

The authors declare that the data supporting the findings of this study are available within the paper. Should any raw data files be needed in another format, they are available from the corresponding author upon reasonable request. All code for data analysis associated with the current submission is available at https://github.com/github-fxw/Feng.et-al.ijms (accessed on 16 April 2024).

## Supplementary Materials

The following supporting information can be downloaded at https://www.mdpi.com/article/10.3390/ijms25094621/s1.

## Abbreviations

AXL: AXL Receptor Tyrosine Kinase; AD: Alzheimer’s disease; BACE1: Beta-secretase 1; BDNF: Brain-derived neurotrophic factor; CD44: Cluster of Differentiation 44; CLEC4G: C-type lectin domain family 4 member G; CLEC5A: C-type lectin domain family 5 member A; COLEC12: Collectin Subfamily Member 12; DAP12: DNAX-activating protein 12; FGL1: Fibrinogen-Like 1; GTPBP1: GTP-Binding Protein 1; ICAM2: Intercellular Adhesion Molecule 2; ICAM3: Intercellular Adhesion Molecule 3; iPSC: Induced Pluripotent Stem Cells; LAG3: Lymphocyte-Activating 3; MGAT1: Mannosyl (Al-pha-1,3-)-Glycoprotein Beta-1,2-N-Acetylglucosaminyltransferase; MGAT3: Mannosyl (Be-ta-1,4-)-Glycoprotein Beta-1,4-N-Acetylglucosaminyltransferase; NGF: Nerve Growth Factor; NPCs: Neural progenitor cells; NR2F6: Nuclear Receptor Subfamily 2 Group F Member 6; PHA: Phytohemagglutinin; PILRA: Paired Immunoglobin Like Type 2 Receptor Alpha; SYK: Spleen Associated Tyrosine Kinase; SCENIC: Single-Cell Regulatory Network Inference and Clustering; scRNA-seq: Single-cell RNA sequencing; TRAPPC5: Trafficking Protein Particle Complex Subunit 5; TYRO3: TYRO3 Protein Tyrosine Kinase; TYROBP: Tyrosine-based Activation Motif-Bearing Adapter.

## References

[1] Zhang Y., Ren R., Yang L., Zhang H., Shi Y., Okhravi H.R., Vitiello M.V., Sanford L.D., Tang X. (2022). Sleep in Alzheimer’s Disease: A Systematic Review and Meta-Analysis of Polysomnographic Findings. Transl. Psychiatry.

[2] Volloch V., Rits-Volloch S. (2023). Next Generation Therapeutic Strategy for Treatment and Prevention of Alzheimer’s Disease and Aging-Associated Cognitive Decline: Transient, Once-in-a-Lifetime-Only Depletion of Intraneuronal Aβ (iAβ) by Its Targeted Degradation via Augmentation of Intra-iAβ-Cleaving Activities of BACE1 and/or BACE2. Int. J. Mol. Sci..

[3] Volloch V., Rits-Volloch S. (2023). The Amyloid Cascade Hypothesis 2.0 for Alzheimer’s Disease and Aging-Associated Cognitive Decline: From Molecular Basis to Effective Therapy. Int. J. Mol. Sci..

[4] Tang L., Yang J., Tang X., Ying W., Qian X., He F. (2010). The DC-SIGN Family Member LSECtin Is a Novel Ligand of CD44 on Activated T Cells. Eur. J. Immunol..

[5] Yang Z., Li Q., Wang X., Jiang X., Zhao D., Lin X., He F., Tang L. (2018). C-Type Lectin Receptor LSECtin-Mediated Apoptotic Cell Clearance by Macrophages Directs Intestinal Repair in Experimental Colitis. Proc. Natl. Acad. Sci. USA.

[6] Rahimi N. (2020). C-Type Lectin CD209L/L-SIGN and CD209/DC-SIGN: Cell Adhesion Molecules Turned to Pathogen Recognition Receptors. Biology.

[7] Brown G.D., Willment J.A., Whitehead L. (2018). C-Type Lectins in Immunity and Homeostasis. Nat. Rev. Immunol..

[8] Wohlfeil S.A., Häfele V., Dietsch B., Schledzewski K., Winkler M., Zierow J., Leibing T., Mohammadi M.M., Heineke J., Sticht C. (2019). Hepatic Endothelial Notch Activation Protects against Liver Metastasis by Regulating Endothelial-Tumor Cell Adhesion Independent of Angiocrine Signaling. Cancer Res..

[9] Kizuka Y., Kitazume S., Sato K., Taniguchi N. (2015). Clec4g (LSECtin) Interacts with BACE1 and Suppresses Aβ Generation. FEBS Lett..

[10] Moreno-Jiménez E.P., Flor-García M., Terreros-Roncal J., Rábano A., Cafini F., Pallas-Bazarra N., Ávila J., Llorens-Martín M. (2019). Adult Hippocampal Neurogenesis Is Abundant in Neurologically Healthy Subjects and Drops Sharply in Patients with Alzheimer’s Disease. Nat. Med..

[11] Terreros-Roncal J., Flor-García M., Moreno-Jiménez E.P., Rodríguez-Moreno C.B., Márquez-Valadez B., Gallardo-Caballero M., Rábano A., Llorens-Martín M. (2023). Methods to Study Adult Hippocampal Neurogenesis in Humans and across the Phylogeny. Hippocampus.

[12] Meng Y., Heybrock S., Neculai D., Saftig P. (2020). Cholesterol Handling in Lysosomes and Beyond. Trends Cell Biol..

[13] Rogers M.A., Chang C.C., Maue R.A., Melton E.M., Peden A.A., Garver W.S., Lee J., Schroen P., Huang M., Chang T.-Y. (2022). Acat1/Soat1 Knockout Extends the Mutant Npc1 Mouse Lifespan and Ameliorates Functional Deficiencies in Multiple Organelles of Mutant Cells. Proc. Natl. Acad. Sci. USA.

[14] Kågedal K., Kim W.S., Appelqvist H., Chan S., Cheng D., Agholme L., Barnham K., McCann H., Halliday G., Garner B. (2010). Increased Expression of the Lysosomal Cholesterol Transporter NPC1 in Alzheimer’s Disease. Biochim. Biophys. Acta (BBA)-Mol. Cell Biol. Lipids.

[15] Li D., Zhang J., Liu Q. (2022). Brain Cell Type-Specific Cholesterol Metabolism and Implications for Learning and Memory. Trends Neurosci..

[16] Haure-Mirande J.-V., Audrain M., Ehrlich M.E., Gandy S. (2022). Microglial TYROBP/DAP12 in Alzheimer’s Disease: Transduction of Physiological and Pathological Signals across TREM2. Mol. Neurodegener..

[17] Smith A.M., Davey K., Tsartsalis S., Khozoie C., Fancy N., Tang S.S., Liaptsi E., Weinert M., McGarry A., Muirhead R.C.J. (2022). Diverse Human Astrocyte and Microglial Transcriptional Responses to Alzheimer’s Pathology. Acta Neuropathol..

[18] Audrain M., Haure-Mirande J.-V., Mleczko J., Wang M., Griffin J.K., St George-Hyslop P.H., Fraser P., Zhang B., Gandy S., Ehrlich M.E. (2021). Reactive or Transgenic Increase in Microglial TYROBP Reveals a TREM2-Independent TYROBP-APOE Link in Wild-Type and Alzheimer’s-Related Mice. Alzheimers Dement..

[19] Tang X., Tena J., Di Lucente J., Maezawa I., Harvey D.J., Jin L.-W., Lebrilla C.B., Zivkovic A.M. (2023). Transcriptomic and Glycomic Analyses Highlight Pathway-Specific Glycosylation Alterations Unique to Alzheimer’s Disease. Sci. Rep..

[20] Kizuka Y., Kitazume S., Taniguchi N. (2017). N-Glycan and Alzheimer’s Disease. Biochim. Biophys. Acta (BBA)-Gen. Subj..

[21] Biswas B., Batista F., Sundaram S., Stanley P. (2018). MGAT1 and Complex N-Glycans Regulate ERK Signaling during Spermatogenesis. Sci. Rep..

[22] Aibar S., González-Blas C.B., Moerman T., Huynh-Thu V.A., Imrichova H., Hulselmans G., Rambow F., Marine J.-C., Geurts P., Aerts J. (2017). SCENIC: Single-Cell Regulatory Network Inference and Clustering. Nat. Methods.

[23] Zhang F., Ren S., Zuo Y. (2014). DC-SIGN, DC-SIGNR and LSECtin: C-Type Lectins for Infection. Int. Rev. Immunol..

[24] Monteiro J.T., Lepenies B. (2017). Myeloid C-Type Lectin Receptors in Viral Recognition and Antiviral Immunity. Viruses.

[25] Rebelo A.L., Chevalier M.T., Russo L., Pandit A. (2022). Role and Therapeutic Implications of Protein Glycosylation in Neuroinflammation. Trends Mol. Med..

[26] Kizuka Y., Taniguchi N. (2018). Neural Functions of Bisecting GlcNAc. Glycoconj. J..

[27] Kizuka Y., Kitazume S., Fujinawa R., Saito T., Iwata N., Saido T.C., Nakano M., Yamaguchi Y., Hashimoto Y., Staufenbiel M. (2015). An Aberrant Sugar Modification of BACE 1 Blocks Its Lysosomal Targeting in A Lzheimer’s Disease. EMBO Mol. Med..

[28] Hodges A.K., Piers T.M., Collier D., Cousins O., Pocock J.M. (2021). Pathways Linking Alzheimer’s Disease Risk Genes Expressed Highly in Microglia. Neuroimmunol. Neuroinflamm..

[29] Andrade-Guerrero J., Santiago-Balmaseda A., Jeronimo-Aguilar P., Vargas-Rodríguez I., Cadena-Suárez A.R., Sánchez-Garibay C., Pozo-Molina G., Méndez-Catalá C.F., Cardenas-Aguayo M.-C., Diaz-Cintra S. (2023). Alzheimer’s Disease: An Updated Overview of Its Genetics. Int J Mol Sci.

[30] Mossad O., Erny D. (2020). The Microbiota–Microglia Axis in Central Nervous System Disorders. Brain Pathol..

[31] Wang S., Sudan R., Peng V., Zhou Y., Du S., Yuede C.M., Lei T., Hou J., Cai Z., Cella M. (2022). TREM2 Drives Microglia Response to Amyloid-β via SYK-Dependent and -Independent Pathways. Cell.

[32] Zhao D., Han X., Zheng X., Wang H., Yang Z., Liu D., Han K., Liu J., Wang X., Yang W. (2016). The Myeloid LSECtin Is a DAP12-Coupled Receptor That Is Crucial for Inflammatory Response Induced by Ebola Virus Glycoprotein. PLoS Pathog..

[33] Bettcher B.M., Tansey M.G., Dorothée G., Heneka M.T. (2021). Peripheral and Central Immune System Crosstalk in Alzheimer Disease—A Research Prospectus. Nat. Rev. Neurol..

[34] Kummer M.P., Hammerschmidt T., Martinez A., Terwel D., Eichele G., Witten A., Figura S., Stoll M., Schwartz S., Pape H.-C. (2014). Ear2 Deletion Causes Early Memory and Learning Deficits in APP/PS1 Mice. J. Neurosci..

[35] He F., Liu Z., Xu J., Xiong Y., Zhang X., Qi J., Lin X., Chu C., Shen L., Liu G. (2023). Black Phosphorus Nanosheets Suppress Oxidative Damage of Stem Cells for Improved Neurological Recovery. Chem. Eng. J..

[36] Quinn T.P., Crowley T.M., Richardson M.F. (2018). Benchmarking Differential Expression Analysis Tools for RNA-Seq: Normalization-Based vs. Log-Ratio Transformation-Based Methods. BMC Bioinform..

[37] Wang Z., Young R.L., Xue H., Wagner G.P. (2011). Transcriptomic Analysis of Avian Digits Reveals Conserved and Derived Digit Identities in Birds. Nature.

[38] Szklarczyk D., Kirsch R., Koutrouli M., Nastou K., Mehryary F., Hachilif R., Gable A.L., Fang T., Doncheva N.T., Pyysalo S. (2023). The STRING Database in 2023: Protein-Protein Association Networks and Functional Enrichment Analyses for Any Sequenced Genome of Interest. Nucleic Acids Res..

[39] Doncheva N.T., Morris J.H., Gorodkin J., Jensen L.J. (2019). Cytoscape StringApp: Network Analysis and Visualization of Proteomics Data. J. Proteome Res..

