# Supplementary material for: Reduced Expression of CLEC4G in Neurons Is Associated with Alzheimer’s Disease
Source: Int J Mol Sci. 2024 Apr 24;25(9):4621. doi: 10.3390/ijms25094621 (PMC11083414; doi:10.3390/ijms25094621)
Supplement: Supplementary file 1 [file ijms-25-04621-s001.zip › ijms-2933841-supplementary.pdf]

Supplementary Materials

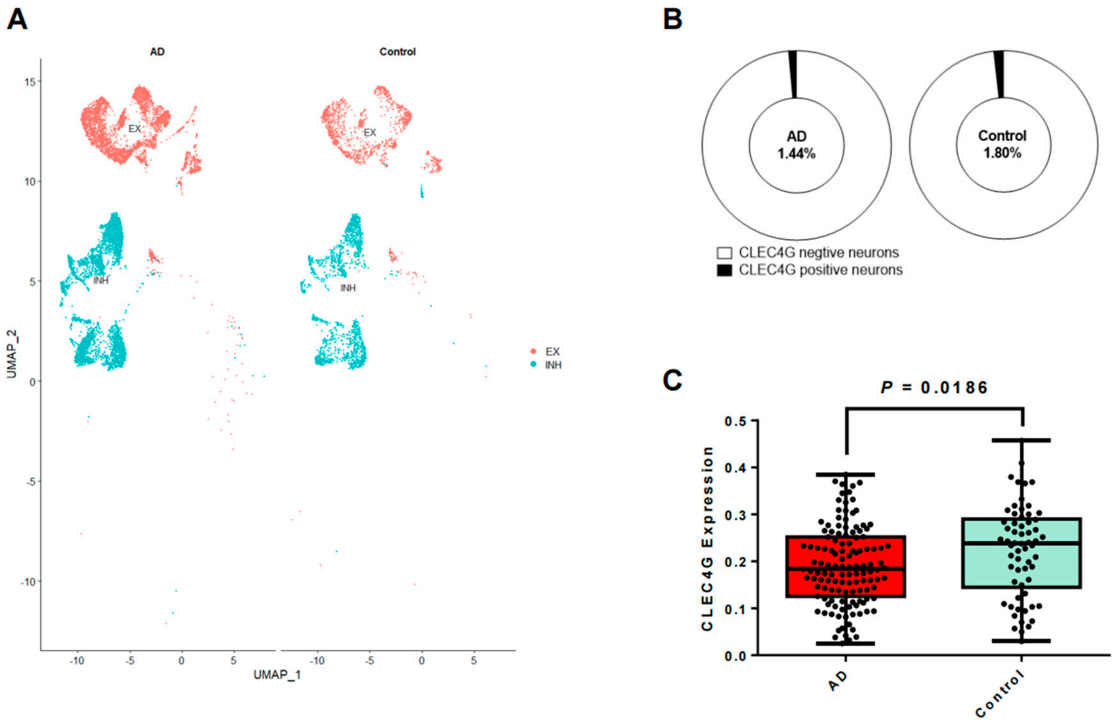

Figure S1. Analysis of CLEC4G expression in neuronal cell single-cell data. (A) UMAP visualization of excitatory neurons (EX) and inhibitory neurons (INH) in AD and control groups. (B) CLEC4G expression ratio in neurons of the AD and control groups. (scRNA-seq data source: GSE174367) (C) CLEC4G expression level in the AD and control groups.

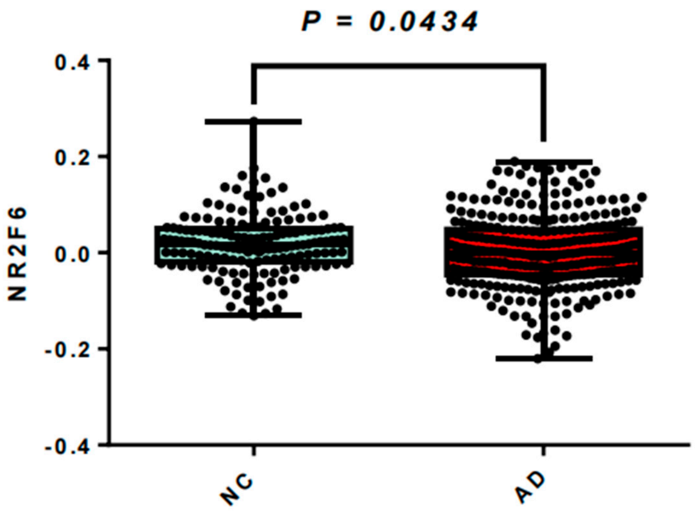

Figure S2. Comparison of NR2F6 Expression Levels between Normal (NC) and Alzheimer's Disease (AD) Groups. Data resource: GSE33000 Dataset from GEO database.

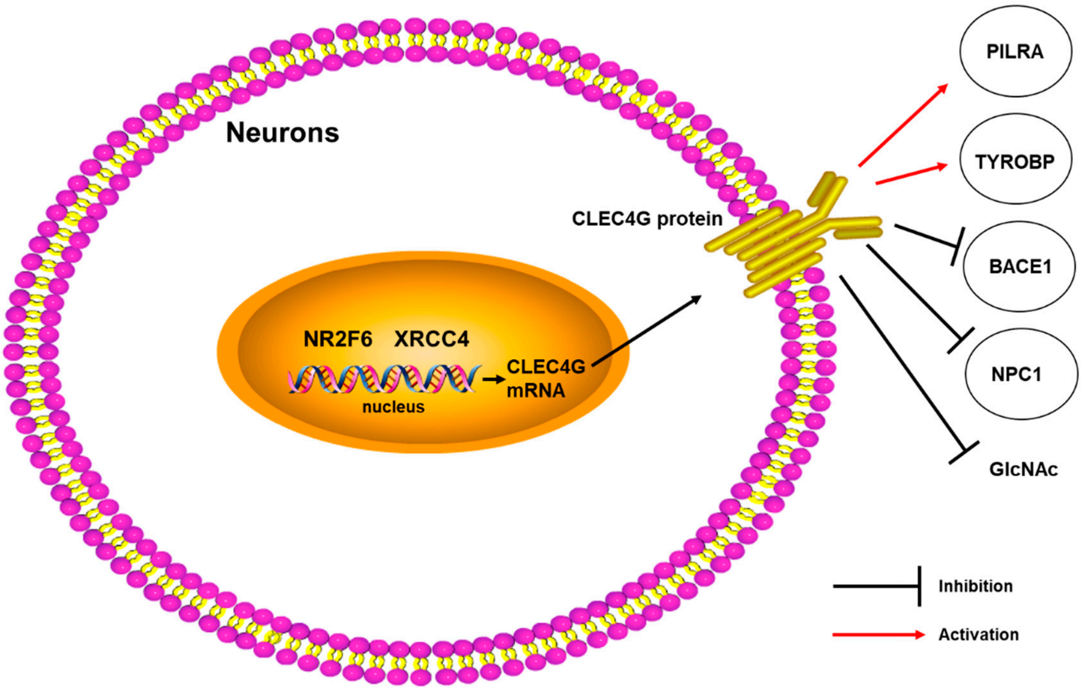

Figure S3. Schematic figure of hypothetical regulatory pathway of CLEC4G in neurons.

Table S1. Details of GEO series included in this analysis

GEO series	Contributor, Year	Alzheimer's disease	Non-demented	Platform
GSE33000	Narayanan M et al, 2014	310	157	Rosetta/Merck Human 44k 1.1 microarray
GSE39420	Antonell A et al, 2015	14	7	[HuGene-1_1-st] Affymetrix Human Gene 1.1 ST Array
GSE174367	Morabito S et al, 2021	38676 (nuclei)	22796 (nuclei)	Illumina NovaSeq 6000

Table S2. Detailed culture medium formulation

Medium	Components	Manufacturer	Concentration
NIM1	DMEM/F12	Gibco	50%
	Neurobasal	Gibco	50%
	N2	STEMCELL	1X
	B27	STEMCELL	1X
	hLIF	PEPROTech	10 ng/ml
	SB431542	Med Chem Express	2 µM
	CHIR99021	Med Chem Express	3 µM
	Compound E	Med Chem Express	0.1 µM

NIM2	DMEM/F12	Gibco	50%
	Neurobasal	Gibco	50%
	N2	STEMCELL	1X
	B27	STEMCELL	1X
	hLIF	PEPROTech	10 ng/ml
	SB431542	Med Chem Express	4 μM
	CHIR99021	Med Chem Express	3 μM
	Compound E	Med Chem Express	0.1 μM
NSMM	DMEM/F12	Gibco	50%
	Neurobasal	Gibco	50%
	N2	STEMCELL	1X
	B27	STEMCELL	1X
	hLIF	PEPROTech	10 ng/ml
	SB431542	Med Chem Express	2 μM
	CHIR99021	Med Chem Express	3 μM
DM	DMEM/F12	Gibco	
	N2	STEMCELL	1X

B27	STEMCELL	1X
dbcAMP	Sigma	400 μM
ascorbic acid	Sigma	200 μM
BDNF	PEPROTech	10 ng/ml
NGF	PEPROTech	10 ng/ml
